# Estimating the number of usability problems affecting medical devices: modelling the discovery matrix

**DOI:** 10.1186/s12874-020-01091-y

**Published:** 2020-09-18

**Authors:** Vincent Vandewalle, Alexandre Caron, Coralie Delettrez, Renaud Périchon, Sylvia Pelayo, Alain Duhamel, Benoit Dervaux

**Affiliations:** 1 Univ. Lille, CHU Lille, ULR 2694 Evaluations des technologies de santé et des pratiques médicales, F-59000 Lille, France; 2 Inria, F-59000 Lille, France; 3 CHU Lille, Direction de la Recherche et de l’Innovation, F-59000 Lille, France; 4 Inserm, CIC-IT/Evalab 1403, F-59000 Lille, France

**Keywords:** Usability testing, Medical device, Missing data, Bayesian statistics, Maximum likelihood

## Abstract

**Background:**

Usability testing of medical devices are mandatory for market access. The testings’ goal is to identify usability problems that could cause harm to the user or limit the device’s effectiveness. In practice, human factor engineers study participants under actual conditions of use and list the problems encountered. This results in a binary discovery matrix in which each row corresponds to a participant, and each column corresponds to a usability problem. One of the main challenges in usability testing is estimating the total number of problems, in order to assess the completeness of the discovery process. Today’s margin-based methods fit the column sums to a binomial model of problem detection. However, the discovery matrix actually observed is truncated because of undiscovered problems, which corresponds to fitting the marginal sums without the zeros. Margin-based methods fail to overcome the bias related to truncation of the matrix. The objective of the present study was to develop and test a matrix-based method for estimating the total number of usability problems.

**Methods:**

The matrix-based model was based on the full discovery matrix (including unobserved columns) and not solely on a summary of the data (e.g. the margins). This model also circumvents a drawback of margin-based methods by simultaneously estimating the model’s parameters and the total number of problems. Furthermore, the matrix-based method takes account of a heterogeneous probability of detection, which reflects a real-life setting. As suggested in the usability literature, we assumed that the probability of detection had a logit-normal distribution.

**Results:**

We assessed the matrix-based method’s performance in a range of settings reflecting real-life usability testing and with heterogeneous probabilities of problem detection. In our simulations, the matrix-based method improved the estimation of the number of problems (in terms of bias, consistency, and coverage probability) in a wide range of settings. We also applied our method to five real datasets from usability testing.

**Conclusions:**

Estimation models (and particularly matrix-based models) are of value in estimating and monitoring the detection process during usability testing. Matrix-based models have a solid mathematical grounding and, with a view to facilitating the decision-making process for both regulators and device manufacturers, should be incorporated into current standards.

## Background

### Introduction

The usability testing is a cornerstone of medical device development, and proof of usability is mandatory for market access in both the European Union and the United States [1]. The overall objective of a usability assessment is to ensure that a medical device is designed and optimized for use by the intended users in the environment in which the device is likely to be used [2]. The goal is to identify problems (called “use errors”) that could cause harm to the user or impair medical treatment (e.g. an inappropriate number of inhalations, finger injection with an adrenaline pen, etc.) [3]. The detection of usability problems must be as comprehensive as possible because medical devices are safety-critical systems [4]. However, the total number of usability problems is never known in advance. The main challenge during the usability testing is thus to estimate this number, in order to assess the completeness of the problem discovery process [5].

In practice, participants are placed under actual conditions of use (real or simulated), and usability problems are observed and listed by human factor engineers. The experimental conditions are defined in a risk analysis that gathers together possible usability problems. Throughout the usability testing, problems are discovered and added to a discovery matrix - a binary matrix with the participants as the rows and the problems as the columns. The current approach involves estimating the total number of problems as the usability testing progresses, starting from the first sessions. The number is estimated iteratively as the sample size increases, until the objective of completeness has been achieved [6].

From a statistical perspective, the current estimation procedure is based on a model of how the usability problems are detected; this is considered to be a binomial process. The literature suggests that the total number of usability problems can be estimated from the discovery matrix’s problem margin (the sum of the columns) [7–11]. However, this estimation is complicated by (i) the small sample size usually encountered in usability testing of medical devices [12] and (ii) as-yet unobserved problems that truncate the margin and bias estimates [13–15].

The objective of the present study was to develop a matrix-based estimation of the number of usability problems affecting a medical device. This new method is based on the likelihood of the discovery matrix (rather than the matrix’s margins alone), so as to avoid a reduction in the level of information prior to modeling. The method’s main targets are (i) regulatory agencies and notified bodies involved in the pre-market evaluation of medical devices, and (ii) medical device manufacturers (more specifically, the human factors engineers in charge of ensuring that the devices are usable).

### Data collected during the usability testing: the discovery matrix

The human factor engineer collects the results of the usability testing in a problem-discovery matrix $\mathbbm{d}$. Each row corresponds to a participant, and each column corresponds to a usability problem. The result is 1 if the participant discovered the problem and 0 if not. Considering that after the inclusion of *n* participants, *j* problems have been discovered, a *n* × *j* matrix is built. By way of an example, the discovery matrix obtained after *n* = 8 participants (in rows) might be the one presented below:

$$ \mathbbm{d}=\left(\begin{array}{cccccccccc}1& 0& 0& 0& 0& 0& 0& 0& 0& 0\\ {}0& 1& 1& 0& 0& 0& 0& 0& 0& 0\\ {}0& 0& 0& 1& 1& 1& 1& 0& 0& 0\\ {}1& 0& 0& 0& 0& 0& 0& 1& 0& 0\\ {}0& 0& 0& 1& 0& 0& 0& 1& 1& 0\\ {}0& 1& 0& 0& 0& 0& 0& 0& 0& 1\\ {}0& 0& 0& 0& 1& 0& 0& 0& 0& 1\\ {}0& 0& 1& 0& 0& 1& 0& 0& 0& 0\end{array}\right) $$

In this example, *j* = 10 different problems (in columns) have been detected so far. The first participant discovered only one problem (column 1), whereas the second discovered two new problems (columns 2 and 3), etc.

At this stage, some problems might not have been detected, and the total number of usability problems (*m*) is unknown. It should be noted that by definition, *m* ≥ *j* and *m* − *j* problems remain undetected. Indeed, $\mathbbm{d}$ comes from a complete but unobserved matrix of dimensions *n* × *m*. This matrix is denoted as $\mathbbm{x}$. Thus, the “observed” matrix $\mathbbm{d}$ is a truncated version of the “complete” matrix $\mathbbm{x}$; it lacks the columns corresponding to the as-yet undetected problems. Hereafter, we use the following notation: $\mathbbm{x}={\left({x}_{il}\right)}_{1\le i\le n,1\le l\le m}$ where *x*_*il*_ = 1 if the participant *i* experiences the problem *l*, and *x*_*il*_ = 0 otherwise.

$$ \mathbbm{x}=\left(\begin{array}{ccccc}{x}_{11}& \cdots & {x}_{1l}& \cdots & {x}_{1m}\\ {}\vdots & \ddots & \vdots & \ddots & \vdots \\ {}{x}_{i1}& \cdots & {x}_{il}& \cdots & {x}_{im}\\ {}\vdots & \ddots & \vdots & \ddots & \vdots \\ {}{x}_{n1}& \cdots & {x}_{nl}& \cdots & {x}_{nm}\end{array}\right) $$

The human factor engineer’s goal is to estimate the total number of problems *m* from the discovery matrix $\mathbbm{d}$ and thus deduce *m* − *j* - the number of problems that have not been detected. The new method presented below addresses this goal.

### Conventional estimation of *m* using a margin-based probabilistic model

In this section, we describe the margin-based methods currently employed to estimate the number of usability problems. As mentioned above, *m* is currently estimated by fitting a probabilistic (binomial) model to the discovery matrix’s problems margin. More specifically, the probability with which a given usability problem is discovered by a participant is modelled by a Bernoulli trial with a probability of success (i.e. detection) *p*. For a given problem, the Bernoulli trial is considered to apply independently to each of the *n* participants in the usability testing. Thus, the problem margin sums can be considered as an independent, identically distributed sequence of Bernoulli trials, in which the number of times a given usability problem (a random variable *X*) has been observed after *n* participants follows a binomial distribution, $X\sim \mathcal{B} in\left(n,p\right)$. Considering the binomial distribution of the margin sums, the proportion of problems that has been discovered at least once after *n* participants is given by the cumulative function of the shifted geometric distribution [6, 16, 17]:

1 $$ P\left(X>0\right)=1-{\left(1-p\right)}^n $$

The total number of problems *m* is then deduced from the following relationship:

2 $$ j=\left(1-{\left(1-p\right)}^n\right)\times m $$

The discovery progress is thus assessed in two steps: the probability of detection *p* is first estimated and then plugged into Eq. (2) to estimate the number of problems *m*. A wide range of literature methods are available for estimating the probability of problem detection. The simplest way involves computing the naive estimate (denoted as $\hat{p}$) using the observed discovery matrix $\mathbbm{d}$, considering that only *j* problems have been detected so far:

3 $$ \hat{p}=\frac{\sum_{i=1}^n{\sum}_{l=1}^j{x}_{il}}{n\ast j} $$

As mentioned above, the naïve estimate is systematically biased - especially for small samples. Indeed, unobserved problems result in zero columns that shrink the probability space and lead to overestimation of *p*, particularly at the beginning of the process when *j* ≪ *m*. Consequently, *m* is systematically underestimated, which generates safety concerns in the medical device field. In response, several strategies have been employed to overcome the truncated matrix problem.

In 2001, Hertzum and Jacobsen suggested normalizing the value of $\hat{p}$ [9]. This procedure considers that the lower boundary of the probability of detection estimated with *n* participants is 1/*n*. For example, in a sample of 5 participants, $\hat{p}\in \left[0.2;1\right]$. Conversely, the normalized estimator ${\hat{p}}_{Norm}\in \left[0;1\right]$, and is computed as follows:

4 $$ {\hat{p}}_{\mathrm{Norm}}=\frac{\hat{p}-\frac{1}{n}}{1-\frac{1}{n}} $$

However, the normalized approach suffers from a major limitation when estimating the total number of problems with Eq. (4). In fact, if each participant has discovered only one problem and if each problem was discovered only once, $\hat{p}=\frac{1}{n}$, ${\hat{p}}_{Norm}=0$, and the estimated number of problems $\hat{m}$ is infinite. We will not discuss this estimation method further.

Turing and Good developed a discounting method for estimating the probability of unseen species on the basis of observed data [18]. Lewis suggested that the Good-Turing (GT) adjustment could be used to reduce the magnitude of the overestimation of *p* by increasing the probability space and thus accounting for unobserved usability problems [8]. The GT adjustment is computed as the proportion of singletons relative to the total number of events (i.e. the proportion of problems discovered only once, *x*_*il*_ = 1), and is incorporated in the estimation as follows:

5 $$ {\hat{\mathrm{p}}}_{\mathrm{GT}}=\frac{\hat{p}}{1+ GT} $$

However, Lewis observed that use of the GT estimator overestimated *p*. He empirically assessed the best adjustment for a small sample size by carrying out Monte Carlo simulations on a range of usability testing databases involving web or software user interfaces with known true values. Based on these simulations, Lewis concluded that the best method was to average the GT adjustment and a “double-deflation” term:

6 $$ {\hat{p}}_{\mathrm{double}-\mathrm{deflation}}=\frac{1}{2}\left[\frac{\hat{p}}{1+G{T}_{adj}}\right]+\frac{1}{2}\left[\left(\hat{p}-\frac{1}{n}\right)\times \left(1-\frac{1}{n}\right)\right] $$

Nevertheless, the degree of adjustment of the probability space for unobserved problems is essentially empirical. The residual bias is not known to trend towards over- or underestimation.

In 2009, Schmettow considered the problem margin sums in a zero-truncation framework [19]. Indeed, the distribution of the problems so far observed follows a binomial distribution with only a positive integer as support (i.e. a positive or conditional distribution). The distribution is zero-truncated because problems only appear in the discovery matrix once they have been discovered. The probability is then estimated using standard mathematical techniques, such as the maximum likelihood or moment estimator [20–22]. The probability mass function is:

7 $$ P\left(X=k\right)=\left(\genfrac{}{}{0pt}{}{n}{k}\right){p}^k{\left(1-p\right)}^{n-k} $$

and zero truncation is achieved as follows:

8 $$ P{\left(X=k\right)}_{\mathrm{zt}}=\left\{\begin{array}{cc}0& if\ k=0\\ {}\frac{P\left(X=k\right)}{1-P\left(X=0\right)}& if\ k>0\end{array}\right. $$

The probability of problem discovery is then estimated by using maximum likelihood techniques to fit the marginal sums to the zero-truncated binomial distribution. It should be noted that the expected probability of unobserved problems, Pr(*X* = 0), is deduced from the non-truncated function [19].

### Methods taking account of a heterogeneous problem detection probability

All the methods presented above assume that the probability of detection is the same for all usability problems (i.e., the same *p*). However, this assumption is unrealistic and does not hold true in real-life usability testing. Schmettow showed that overdispersion was frequent in the problem margin sums, reflecting heterogeneity in the probability of detection [23]. Furthermore, erroneously ignoring the presence of heterogeneity by using a single, average value of *p* leads to overestimation of the completeness of the discovery process (Jensen’s inequality) [24]. Schmettow tackled this problem by developing a model that incorporated heterogeneity. The probability of detection was considered to be a random variable, which enabled each problem to have its own probability of detection. Schmettow used the logit-normal distribution as a plugin distribution for the probability of detection. Formally, the logit of the probability of detection follows a normal distribution $\mathcal{N}\left(\mu, \sigma \right)$. In this model, the problem margin sums follows a logit-normal binomial distribution and the probability mass function is:

9 $$ P\left(X=k\right)=\left(\genfrac{}{}{0pt}{}{n}{k}\right)\frac{1}{\sqrt{2\pi}\sigma}\underset{0}{\overset{1}{\int }}{\left(1-p\right)}^{n-k-1}{p}^{k-1}\exp \left(-\frac{{\left( logit(p)-\mu \right)}^2}{2{\sigma}^2}\right) dp $$

Using the zero truncation technique presented in eq. (8), Schmettow developed the logit-normal binomial zero truncated (LNBzt) model and applied it to the usability of medical infusion pumps [25]. To the best of our knowledge, this model is the only one that accounts for both heterogeneity and unobserved problems.

### Statistical limitations of margin-based methods

The primary limitation of the margin-based methods presented above is that they estimate the probability of detection only. The number of problems *m* is deduced but not estimated per se. It would be possible to estimate both *m* and *p* by summarizing the discovery matrix on the basis of the participants’ margin. In such a case, each sum follows a binomial $\mathcal{B} in\left(m,p\right)$, thus enabling estimation of both the number of attempts and the probability of success in a binomial setting. However, DasGupta and Rubin established that there were no unbiased estimates for essentially any functions of either the number of attempts or the probability of success [26]. This problem was initially considered by Fisher and Haldane for estimating species abundance [27, 28]. It has also been considered by Olkin, Petkau, and Zidek, who developed both a moment and a maximum likelihood estimator, and by Carroll and Lombard, who proposed an estimator in a Bayesian setting (leading to a beta-binomial distribution) [29, 30]. Hall also considered this problem in an asymptotic framework [31].

The second limitation of margin-based methods is information loss, relative to the initially available data. For example, *j* and the number of singletons were the only data used in the GT estimates. In the same way, the zero-truncated method considered only the column sums for the problems and omitted the pattern of detection (i.e., the users).

Here, we tackle these problems by directly modelling the full discovery matrix (including unobserved columns) and not only a summary of the data (e.g. the margins). In the Methods section, we describe the statistical basis of the matrix-based method and detail a Bayesian approach for estimating the number of problems. In the Results section, we compare the matrix-based method’s statistical properties with those of existing models in a simulation study and then in actual usability studies. Lastly, we discuss the implications of our results with regard to estimation of the number of problems in usability testing.

## Methods

We first specify the statistical basis underpinning the matrix-based method, and the principle of column permutation in particular. Next, we present our estimation of the number of problems in a Bayesian setting. The last part is dedicated to the methods used to assess the matrix-based model’s performance.

### The matrix-based method

We first present the matrix-based method. For the sake of clarity, we simplified the problem by considering that the probability of problem detection was homogeneous. The concept of heterogeneous probability will be introduced in the second part of this section, along with the Bayesian estimation.

### Presentation of the method

Consider the complete discovery matrix $\mathbbm{x}$. The probability of $\mathbbm{x}$ can be written as follows:

10 $$ P\left(\mathbbm{x}|p,m\right)={p}^{{\mathbbm{x}}_{\bullet \bullet }}{\left(1-p\right)}^{nm-{\mathbbm{x}}_{\bullet \bullet }} $$

where ${\mathbbm{x}}_{\bullet \bullet }={\sum}_{i=1}^n{\sum}_{l=1}^m{x}_{il}$ is the total number of problems observed by *n* participants.

An example of a possible matrix $\mathbbm{x}$ obtained from two participants during a usability testing of a medical device with *m* = 3 problems is given below (with users in rows and problems in columns):

11 $$ \mathbbm{x}=\left(\begin{array}{ccc}0& 0& 1\\ {}1& 0& 0\end{array}\right) $$

As seen above, the complete discovery matrix $\mathbbm{x}$ is never observed, and the discovery matrix $\mathbbm{d}$ is the only one available. It is similar to the matrix $\mathbbm{x}$, except that unobserved problems are missing. Considering the above example, neither of the users observed the second problem, and the resulting observed discovery matrix $\mathbbm{d}$ would be:

12 $$ \mathbbm{d}=\left(\begin{array}{cc}1& 0\\ {}0& 1\end{array}\right) $$

It should be noted that if the total number of problems *m* is known, then the complete matrix $\mathbbm{x}$ could be reconstituted (with permutation), based on the matrix $\mathbbm{d}$. For instance, if we take the matrix $\mathbbm{x}$ and consider (wrongly, in this case) that the number of problems *m* = 5, then the reconstituted complete matrix denoted by ${\hat{\mathbbm{x}}}^m$ would be obtained by padding the matrix $\mathbbm{d}$ with columns of zeros (corresponding to as-yet unobserved problems):

13 $$ {\hat{\mathbbm{x}}}^{m=5}=\left(\begin{array}{ccccc}1& 0& 0& 0& 0\\ {}0& 1& 0& 0& 0\end{array}\right) $$

Thus, noting that ${\mathbbm{x}}_{\bullet \bullet }={\mathbbm{d}}_{\bullet \bullet }$, it is possible to compute the likelihood of the complete matrix ${\hat{\mathbbm{x}}}^m$ on the basis of the discovery matrix $\mathbbm{d}$. This likelihood is given by the following equation:

14 $$ P\left({\hat{\mathbbm{x}}}^m|p,m\right)={p}^{{\mathbbm{x}}_{\bullet \bullet }}{\left(1-p\right)}^{nm-{\mathbbm{x}}_{\bullet \bullet }} $$

Note that the definition of ${\hat{\mathbbm{x}}}^m$ depends on the value *m*, which is unknown. Thus, any inference based on ${\hat{\mathbbm{x}}}^m$ will induce some bias. For instance, a maximum likelihood estimation of (*p*, *m*) based on ${\hat{\mathbbm{x}}}^m$ (consisting in maximizing $p\left({\hat{\mathbbm{x}}}^m|p,m\right)$ with respect to *m* and *p*) leads to $\hat{m}=j$ (where *j* is the number of problems observed so far) and $p=\frac{{\mathbbm{x}}_{\bullet \bullet }}{nj}$, which are known to be biased. We tackled this issue by modeling the distribution of the observed discovery matrix $p\left(\mathbbm{d}|p,m\right)$.

It should be noted that the matrix $\mathbbm{d}$ is defined in a lexicographic order, which simply means that the problems are ordered in the order of detection. For instance, the six possible complete matrices $\mathbbm{x}$ leading to the previous matrix $\mathbbm{d}$ if *m* = 3 are presented in Table 1.

**Table 1:** Six possible complete matrices ${\hat{\mathbbm{x}}}^{m=3}$ leading to the observed discovery matrix $$ \mathbbm{d}=\left(\begin{array}{cc}1& 0\\ {}0& 1\end{array}\right) $$

Possibility 1	Possibility 2	Possibility 3
$$ {\hat{\mathbbm{x}}}_1^{m=3}=\left(\begin{array}{ccc}1& 0& 0\\ {}0& 0& 1\end{array}\right) $$	$$ {\hat{\mathbbm{x}}}_2^{m=3}=\left(\begin{array}{ccc}0& 1& 0\\ {}0& 0& 1\end{array}\right) $$	$$ {\hat{\mathbbm{x}}}_3^{m=3}=\left(\begin{array}{ccc}1& 0& 0\\ {}0& 1& 0\end{array}\right) $$
Possibility 4	Possibility 5	Possibility 6
$$ {\hat{\mathbbm{x}}}_4^{m=3}=\left(\begin{array}{ccc}0& 0& 1\\ {}1& 0& 0\end{array}\right) $$	$$ {\hat{\mathbbm{x}}}_5^{m=3}=\left(\begin{array}{ccc}0& 0& 1\\ {}0& 1& 0\end{array}\right) $$	$$ {\hat{\mathbbm{x}}}_6^{m=3}=\left(\begin{array}{ccc}0& 1& 0\\ {}1& 0& 0\end{array}\right) $$

In fact, if we could consider the label (the name of the usability problem) associated with each column, only one matrix $\mathbbm{x}$ could lead to the matrix $\mathbbm{d}$. However, since we have no means of finding the names of the columns in the initial matrix $\mathbbm{x}$, we will consider that the matrix $\mathbbm{d}$ has unnamed columns. Removing these column names allows us to consider the matrix $\mathbbm{d}$ for the observed data (for which the definition does not vary as a function of the model’s definition of the model – in contrast to ${\hat{\mathbbm{x}}}^m$). Thus:

15 $$ P\left(\mathbbm{d}|m=3,p\right)=\sum \limits_{h=1}^6P\Big({\hat{\mathbbm{x}}}_h^{m=3}\left|m=3,p\right) $$

and more generally

16 $$ P\left(\mathbbm{d}|m,p\right)=\sum \limits_{h=1}^{H\left(\mathbbm{d},m\right)}P\Big({\hat{\mathbbm{x}}}_h^m\left|m,p\right) $$

where $H\left(\mathbbm{d},m\right)$ is the number of different matrices ${\hat{\mathbbm{x}}}_h^m$ with *m* columns leading to the same discovery matrix $\mathbbm{d}$.

In the simple example presented above (Table 1), $H\left(\mathbbm{d},m\right)=6$ and each matrix ${\hat{\mathbbm{x}}}_h^m$ has the same probability, i.e. *p*^2^(1 − *p*)^4^. It follows that:

17 $$ {\displaystyle \begin{array}{c}P\left(\mathbbm{d}|m=3,p\right)=\mathrm{H}\left(\mathbbm{d},\mathrm{m}=3\right)\times P\Big({\hat{\mathbbm{x}}}_h^{m=3}\left|m=3,p\right)=\\ {}6\times {p}^2{\left(1-p\right)}^4={A}_3^2\times {p}^2{\left(1-p\right)}^4\end{array}} $$

More generally, the number of matrices $\mathbbm{x}$ with *m* columns associated with an observed discovery matrix $\mathbbm{d}$ is:

18 $$ \mathrm{H}\left(\mathbbm{d},\mathrm{m}\right)=\frac{m!}{\left(m-j\right)!{j}_1!\dots {j}_r!}=\frac{1}{j_1!\dots {j}_r!}\times {A}_m^j $$

where *r* is the number of different columns of $\mathbbm{d}$, and *j*_*h*_ (1 ≤ *h* ≤ *r*) is the number of repetitions of the column of type *h*. Of course, *j* = *j*_1_ + ⋯ + *j*_*r*_. Here, we recognize a familiar equation: that associated with the number of anagrams of a word in which each type of column corresponds to a different letter, including the null column (repeated *m* − *j* times).

Lastly, since each matrix ${\hat{\mathbbm{x}}}_h^m$ has the same probability, we obtain the likelihood of $\mathbbm{d}$ as follows:

19 $$ P\left(\mathbbm{d}|p,m\right)=\frac{1}{j_1!\dots {j}_r!}\times {A}_m^j\times P\Big({\hat{\mathbbm{x}}}_h^m\left|m,p\right) $$

In practice, the computation of $\frac{1}{j_1!\dots {j}_r!}$ has no impact on the estimation, since it is the same for all values of *m* and *p*. This result is not limited to the homogenous setting and would remain valid for any probability of $\mathbbm{x}$ with a column-wise exchangeability property.

In the particular case of the homogeneous setting, we obtain:

20 $$ P\left(\mathbbm{d}|p,m\right)=\frac{1}{j_1!\dots {j}_r!}\times {A}_m^j\times {p}^{{\mathbbm{x}}_{\bullet \bullet }}{\left(1-p\right)}^{nm-{\mathbbm{x}}_{\bullet \bullet }} $$

In the homogeneous setting, our matrix-based approach could be extended to perform maximum likelihood inference or Bayesian inference on the parameters. However, as explained above, this setting is unrealistic in practice and so a heterogeneous probability of detection should be considered in the following section.

## Heterogeneity and Bayesian estimation

We considered a heterogeneous probability of detection; i.e. each problem *l* has its own probability of detection *p*_*l*_. In line with Schmettow’s method, we assume that the probabilities of detection are independent and follow a logit-normal distribution, i.e. $\mathrm{logit}\left({p}_l\right)\sim \mathcal{N}\left(\mu, \sigma \right)$. The model’s parameters are *m*, *μ* and *σ*. Note that *p*_1_, …, *p*_*m*_ are considered as latent random variables - like random effects in the mixed model.

Given these parameters, the likelihood of the discovery matrix $\mathbbm{d}$ can be written as

21 $$ P\left(\mathbbm{d}|\mu, \sigma, m\right)={\int}_0^1\dots {\int}_0^1P\left(\mathbbm{d}|{p}_1,\dots, {p}_m,m\right)f\left({p}_1,\dots, {p}_m|\mu, \sigma \right)d{p}_1\dots d{p}_m $$

where *f*(*p*_1_, *p*_2_, …, *p*_*m*_| *μ*, *σ*) is the probability density function of *p*_1_, *p*_2_, …, *p*_*m*_. Given that the columns are exchangeable, we can also write

22 $$ P\left(\mathbbm{d}|\mu, \sigma, m\right)=\frac{1}{j_1!\dots {j}_r!}\times {A}_m^j\times P\Big({\hat{\mathbbm{x}}}_h^m\left|\mu, \sigma, m\right) $$

which will be useful for subsequent computations.

We now consider a Bayesian framework [32] for estimation of the parameters. This framework has good theoretical properties and can include prior knowledge about the problem’s parameters. Indeed, the distribution of the parameters *P*(*μ*, *σ*, *m*) must first be defined. Moreover, assuming the prior independence of *μ*, *σ* and *m*, *P*(*μ*, *σ*, *m*) = *P*(*μ*)*P*(*σ*)*P*(*m*). We assume a prior uniform distribution for *m*:

23 $$ P(m)=\frac{1}{M}\forall m\in \left\{1,\dots, M\ \right\} $$

The value of *M* is the pre-determined upper boundary for *m*, and should be chosen by the human factor engineer according to the expected maximum possible number of problems. To prevent underestimation, a high value should be used. However, if *M* is unnecessarily high, it will lead to an increase in the computing time.

Since our goal here is to estimate the number of problems, our main interest is $P\left(m|\mathbbm{d}\right)$, which is obtained using Bayes’ theorem:

24 $$ P\left(m|\mathbbm{d}\right)=\frac{\mathrm{P}(m)\times P\left(\mathbbm{d}|m\right)}{\sum_{m^{\prime }=1}^M\mathrm{P}\left(m^{\prime}\right)\times P\left(\mathbbm{d}|m^{\prime}\right)} $$

Thus, we need to compute $P\left(\mathbbm{d}|m\right)$ for each possible value of *m* in {1, …, *M*}. This computation requires computation of the integrated likelihood $P\left(\mathbbm{d}|m\right)$, as follows

25 $$ P\left(\mathbbm{d}|m\right)={\int}_0^{+\infty }{\int}_{-\infty}^{+\infty }P\left(\mathbbm{d}|\mu, \sigma, m\right)P\left(\mu \right)P\left(\sigma \right) d\mu d\sigma $$

The choice of prior distributions for *P*(*μ*) and *P*(*σ*) is discussed below. $P\left(\mathbbm{d}|m\right)$ can be computed by approximating this integral with Markov chain Monte Carlo (MCMC) techniques.

Even though $P\left(m|\mathbbm{d}\right)$ is the main quantity of interest, $P\left(\mu |\mathbbm{d}\right)$ and $P\left(\sigma |\mathbbm{d}\right)$ are also of interest because they can be used as prior distributions for future studies; this will decrease the sample size and improve early estimates as part of an early control strategy.

### Computational aspects

From a computational perspective, and since $P\left(\mathbbm{d}|\mu, \sigma, m\right)=\frac{1}{j_1!\dots {j}_r!}\times {A}_m^j\times P\Big({\hat{\mathbbm{x}}}_h^m\left|\mu, \sigma, m\right)$, we will first focus on the computation based on ${\hat{\mathbbm{x}}}_h^m$ and will then deduce the results for $\mathbbm{d}.$

Let now consider the choice of a prior distribution for *μ* and *σ*. Since *μ* and *σ* are Gaussian distribution parameters and in the absence of additional information (e.g. from previous usability studies), we chose the following flat priors:

- $\mu \sim \mathcal{N}\left(0;\mathcal{A}\right)$: a Gaussian distribution with a high variance $\mathcal{A}$, (e.g. $\mathcal{A}={10}^8\Big)$, mimicking a uniform distribution on *ℝ*,
- ${\sigma}^2\sim \mathrm{inv}-{\upchi}_{\nu}^2$: an inverse chi-squared distribution with *ν* degrees of freedom (typically *ν* = 1).

When the data has a Gaussian distribution, choosing the above priors leads to a conjugated posterior distribution. However, a logistic-normal distribution of the probabilities of detection means that conjugacy cannot be obtained. Thus, estimation of the posterior distribution required the use of MCMC methods. This consisted in drawing *μ* and *σ* for each possible value of *m*, *m* ∈ 1, …, *M* according to their posterior distribution $P\left(\mu, \sigma |m,\mathbbm{d}\right)$, and deducing a numerical approximation of $P\left(\mathbbm{d}|m\right)$ from the Monte-Carlo sample. Lastly, $P\left(m|\mathbbm{d}\right)$ was computed using Bayes’ theorem.

For a fixed value of *m*, we consider sampling from $P\Big(\mu, \sigma \left|{\hat{\mathbbm{x}}}_h^m,m\right)$, computing the integrated likelihood $P\Big({\hat{\mathbbm{x}}}_h^m\left|m\right)$ with bridge sampling [33], and deducing $P\left(\mathbbm{d}|m\right)$.

The parameters *μ* and *σ* (given ${\hat{\mathbbm{x}}}_h^m$ and *m*) are sampled using the parameter space augmented by *p*_1_, …, *p*_*m*_, i.e. the discovery probabilities associated with each column of ${\hat{\mathbbm{x}}}_h^m$. Thus, we will now sample from $\mu, \sigma, {p}_1,\dots, {p}_m\mid {\hat{\mathbbm{x}}}_h^m$, using stan software (adaptative Hamiltonian Monte Carlo algorithm).

### Assessment of the performance of the matrix-based method

We compared the performance of five methods (naïve, GT, double-deflation, LNBzt, and matrix-based methods) first in a simulation study and then using literature data from actual usability studies.

### Simulation study

Each simulation consisted in generating an observed discovery matrix $\mathbbm{d}$ from the usability testing of a hypothetical medical device with a known total number of usability problems *m* and a sample size *n*. The probability of detection was normally distributed ($\mathcal{N}\left(\mu, \sigma \right)$) on a logit scale. The combinations of parameters used in the simulations are specified in Table 2. The values were chosen to reflect a wide range of parameters encountered in usability testing of medical devices.

**Table 2:** Combinations of parameters for the simulation testing with homogeneous and heterogeneous probabilities of detection

Parameter	Values
Total number of usability problems	*m* = 20,50,100
Sample size	*n* = 15,20,30,40,50
Probability of problem detection	*μ* = *logit*(0.1), *logit*(0.2) *σ* = 0.5, 1, 2
Number of combinations tested	90

In each setting (i.e. for each combination of *m*, *μ*, *σ* and *n*), we simulated *S* = 2 × 10^4^ complete discovery matrices, ${\mathbbm{x}}_{m,\mu, \sigma, n,i}$, *i* ∈ {1, 2, …, *S*}.. The matrices $\mathbbm{d}$ were obtained by truncation of the zero columns (problems not yet discovered). We averaged the estimates of *m* over the *S* simulations and computed the 95% fluctuation interval (0.025 and 0.975 quantiles). We also calculated the prediction’s root mean square error (RMSE) as the square root of the mean square difference between the predicted and true values of *m*:

26 $$ RMSE(m)=\sqrt{\frac{1}{S}\sum \limits_{i=1}^S{\left(m-{\hat{m}}_i\right)}^2} $$

When the sample is small, little information is available; a tight credible interval might reflect overconfidence rather than a good estimation. Thus, to gauge the level of confidence that human factor engineers can place in each method, we computed the coverage probability. In each setting, this is the proportion of 95% confidence intervals for the simulated ${\hat{m}}_i$ that include the true value of *m*. The confidence intervals for ${\hat{m}}_i$ were computed using 1000 parametric bootstrap repetitions with the parameters $\left({\hat{m}}_i,{\hat{\mu}}_i,{\hat{\sigma}}_i,n\right)$. For the matrix-based method, we were able to directly compute the 95% confidence interval of the posterior distribution of each simulation, which saved substantial computation time.

### Application to actual usability studies

We applied the above-described methods to the discovery matrices of five published usability studies. Four did not involve a medical device: the EDU3D dataset encompassed 119 problems discovered by 20 participants during the evaluation of virtual environments [34], the MACERR dataset encompassed 145 problems discovered by 15 participants during a scenario-driven usability testing of an integrated office system [35], the MANTEL dataset encompassed 30 problems submitted by 76 expert participants evaluating the specifications of a computer program, and the SAVINGS dataset encompassed 48 usability problems discovered by 34 participants on voice response systems MANTEL and SAVINGS comes from the same experiment on heuristic evaluations [36]. These four studies were included because they have been used in important publications in this field [8] and they enabled us to address heterogeneity in the probability of discovery, in particular [23]. The fifth usability testing involved a medical device: INFPUMP encompassed 107 usability problems discovered by 34 participants (intensive care unit nurses and anesthesiologist) evaluating a prototype medical infusion pump [25].

For each of the five datasets, we computed the estimates and the 95% confidence intervals for the final data. When a sufficient number of participants had been included (i.e. for MANTEL, SAVINGS, and INFPUMP), we addressed the change in the estimates as a function of the sample size.

All the analyses were carried out running R software (version 3.6.1) on several servers equipped with 12-core Intel® Xeon® E5–2650 v4 processors (http://hpc.univ-lille.fr/cluster-hpc-htc). The MCMC was performed using the *Stan* library (http://mc-stan.org) via the *rstan* package [37]. The integrated likelihood was obtained using the *bridge_sampler* function of the *bridgesampling* package [38]. In order to facilitate the matrix-based method’s application in practice, a short step-by-step tutorial (see Additional file 1) and the code (see Additional file 2) is provided as supplementary material. A reproducible R code with the data and the simulation study performed in this manuscript is available on GitHub (https://github.com/alexandre-caron/matrix_based-usability). The link to the archived version referenced in this manuscript is available in the “Availability of data and materials” section.

## Results

### The simulation study

The distributions of the probability of detection for each setting are summarized in Table 3. The distribution shifted to a highest average probability of detection when *μ* increased. It is noteworthy that a higher dispersion (*σ*) not only flattened the distribution but also led to an increase in probability of very rare problems.

**Table 3:**
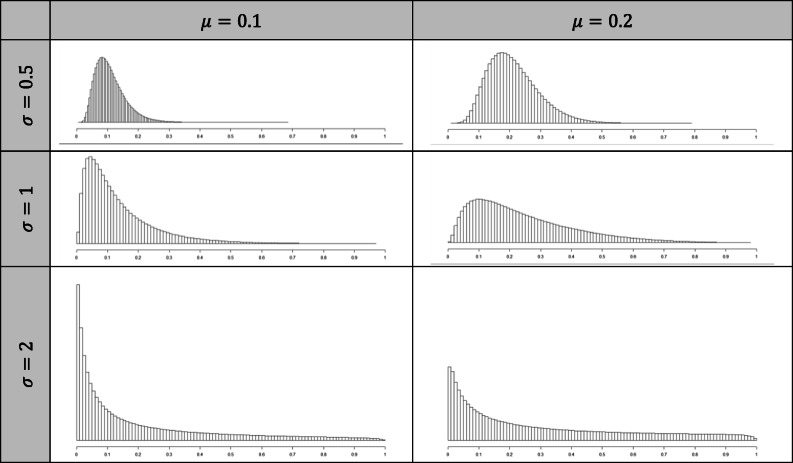
Distribution of the probability of detection as a function of μ and σ. The probability of detection followed a logit-normal distribution: $\mathrm{logit}\left({\mathrm{p}}_{\mathrm{l}}\right)\sim \mathcal{N}\left(\upmu, \upsigma \right)$

**Fig. 1:**
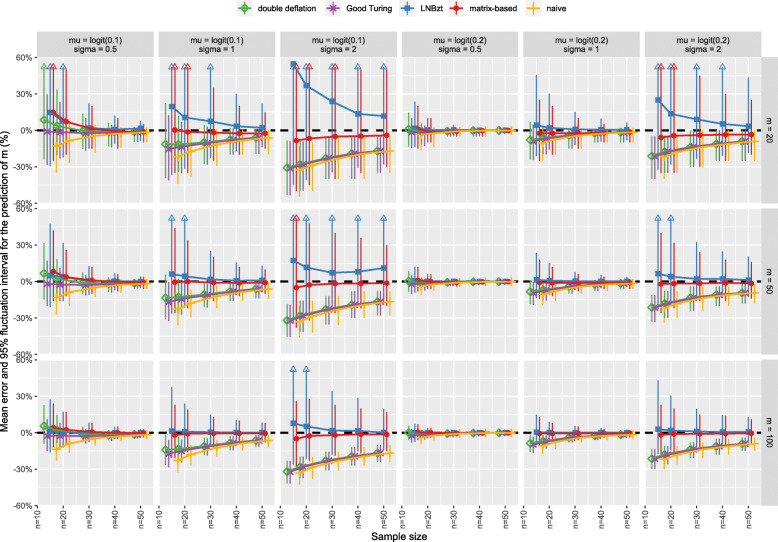
Bias in the prediction of *m*: the mean error and 95% fluctuation interval (as a percentage of the true *m*) as a function of the sample size (*n*). The results are presented for various probabilities of problem detection ((*μ*, *σ*), columns) and various numbers of usability problems (*m*, rows). The dashed line represents the true *m*

**Fig. 2:**
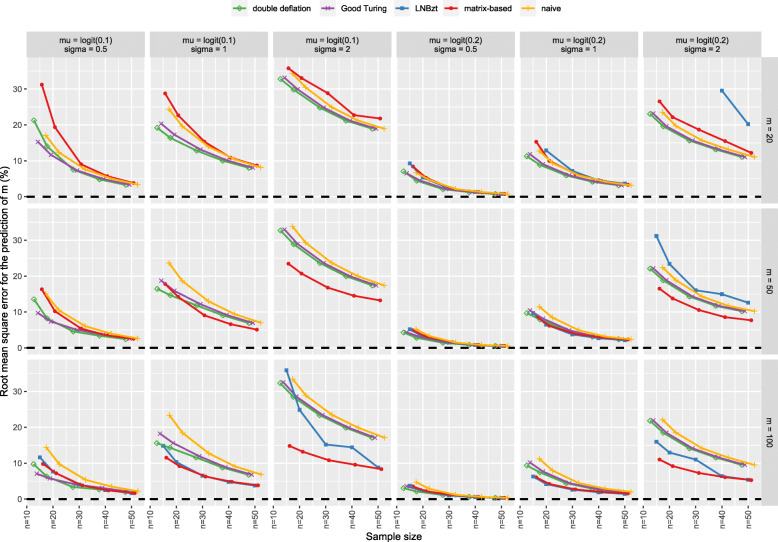
Consistency in the prediction of *m*: the RMSE for the prediction of m (as a percentage of the true *m*) as a function of the sample size (*n*). The results are presented for various probabilities of problem detection ((*μ*, *σ*), columns) and various numbers of usability problems (*m*, rows). The LNBzt results are not represented for *m* < 100 and m *u* = *logit*(0.1), due to a high RMSE

The results of the simulation are presented for the five methods (naïve, GT, double-deflation, LNBzt, and matrix-based). The prediction error of *m* as a function of the sample size *n* are presented in Fig. 1. The RMSE is presented in Fig. 2. A tabulated version of these data is also provided as supplementary material (see S-Table 5 and S-Table 6 in Additional file 2). As mentioned by Schmettow, extreme estimates of *m* can be obtained with the LNBzt method when the number of singletons is high. We decided to discard any results with ${\hat{m}}_{LNBzt}>500$, to avoid penalizing the method with estimates that would not be realistic in real life [19].

As expected, the accuracy of the estimation of the number of problems increased with the sample size for all estimates, with less bias and greater consistency (i.e. the RMSE tended towards zero as the sample size increased). Likewise, the estimates were better as the number of problems to discover *m* increased. For all methods, the bias was higher as the number of “rare” problems increased (i.e. for a higher σ).

#### Methods accounting for heterogeneity: the matrix-based and LNBzt estimates

The matrix-based method showed less bias overall; the bias ranged from − 8.5 to + 14.7% for the 90 simulated combinations. This range was narrower (from − 5.1 to + 1.2%) when the participant sample size was 30 or more. In contrast, the LNBzt method displayed systematic upward bias; although the lower boundary was − 0.1%, the upper boundary was 54.7%. This bias was still observed for 30 participants, with an upper boundary of 23.8%.

When σ = 2, the matrix-based method underestimated the number of problems. However, this underestimation was less than − 5.1% for *n* ≥ 30. For lower values of σ, the matrix-based method’s bias ranged from − 2.6 to + 1.2% for *n* ≥ 30. The bias associated with the LNBzt method was high for σ = 2. Although the bias decreased with *n*, it was still + 11.8% for *n* = 50. For a lower value of σ, the bias associated with the LNBzt method ranged from − 2.6 to + 1.2% for *n* ≥ 30.

The matrix-based method gave the lowest RMSE in all settings. This was particularly true when the number of “rare” problems was high (σ > 0.5). The LNBzt gave the highest average RMSE. As mentioned in the Methods, this bias resulted from a few very high estimates of *m*, which increased the average RMSE dramatically. This was true for the lowest average probability of detection (i.e. μ = logit(0.1)) and the highest variance (i.e. σ = 2).

#### Methods not accounting for heterogeneity: the naïve, GT, and double-deflation estimates

The estimates that did not take account of heterogeneity showed the strongest bias. The naïve estimate was the worst; it systematically underestimated the true value of *m* (range: − 33.2% to − 0.2%). This underestimation was slightly lower for the GT estimate, especially when σ was low. However, the range was still broad: from − 32.2% to − 0.2%. The double-deflation method compensated even more for underestimation but sometimes led to overestimation (range: − 32.0 to + 8.6%).

When σ was lower (i.e. 0.5 or 1), the trend towards underestimation was less pronounced for the double-deflation and the GT methods (with lower boundaries of − 14.1% and − 17.2, respectively) than for the naïve method (lower boundary: − 22.8%. The bias persisted for larger sample sizes: it was still as high as − 6.4% for the three methods for *n* = 50.

The naïve RMSE estimate was again the worst of the methods that did not take account of heterogeneity. Although the GT and the double-deflation methods gave acceptable RMSEs, this feature must be interpreted with caution. In fact, the acceptable RMSEs resulted essentially from systematic underestimation, which in turn limited the range of possible $\hat{m}$ (which can never be lower than *j*). Hence, the interpretation of the RMSE was limited for these methods.

#### Coverage probability

As explained in the Methods, human factor engineers do not know the variables for the usability testing they are carrying out. The coverage probability enables them to study the reliability of the estimate (and its 95% confidence interval). A tabulated version of the data is provided as supplementary material (see S-Table *7* in Additional file 2).

For the matrix-based method, the coverage probability was always over 80% (except for *m* = 100, *n* = 15, *μ* = *logit*(0.1), and *σ* = 0.5, where the probability of coverage dropped to 72%) with an average of 94% over the range of settings tested in the simulations study. The probability was at least 81% for *n* ≥ 20 and at least 88% for *n* ≥ 30. The LNBzt method’s coverage probability was always over 80%, with an average of 92%. The LNBzt performed particularly well for small sample sizes, with a minimum coverage of 89% for *n* = 15, of 86% for *n* = 20, and of 82% for *n* = 30. Indeed, the LNBzt method provided the broadest confidence intervals of the five methods studied here. It is noteworthy that the LNBzt method was the only one that sometimes failed to fit the data (in 33% of cases). However, it was impossible to adjust the method’s parameter for each individual simulation. In practice, changing the optimization function’s starting values would avoid most of the fitting failures.

The methods not taking account of heterogeneity provided a low, erratic coverage probability in most settings. On average, the coverage probabilities were 17.9, 31.5 and 33.7% for the naïve, GT, and double-deflation methods, respectively. Furthermore, the three methods frequently yielded excessively high estimated levels of confidence - especially for high values of *m*.

#### Lessons learned from the simulation study

From the human factor engineer’s point of view, the matrix-based and LNBzt methods are the only reliable ones; they gave a good coverage probability in almost any setting and for almost any sample size. Conversely, the methods not taking account of heterogeneity were unreliable and so could not be trusted.

### Application to real data from published usability studies

The estimated number of problems computed from the discovery matrices of five published usability studies are presented in Table 4. Although the real number of problems is not known, we can compare the matrix-based method’s predictions with those of the other methods (and especially the LNBzt method).

**Table 4:** The estimated number of problems for five real datasets from published usability studies

	n*	j**	naïve	Good-Turing	double deflation	LNBzt	matrix-based
**EDU3D**	**20**	**119**					
$\hat{m}$			120	121	122	155	152
95%CI			117–121	118–125	120–129	132–195	135–167
**MACERR**	**15**	**145**					
$\hat{m}$			156	178	184	449	382
95%CI			146–160	171–207	192–245	256–1301	346–440
**MANTEL**	**76**	**30**					
$\hat{m}$			30	30	30	31	30
95%CI			30–30	30–30	30–30	31–35	30–37
**SAVINGS**	**34**	**44**					
$\hat{m}$			44	44	44	46	45
95%CI			44–45	44–45	44–45	42–50	44–51
**INFPUMP**	**34**	**107**					
$\hat{m}$			107	107	107	122	120
95%CI			107–108	106–108	106–108	110–136	112–143

* *n* is the number of participants in the study

** *j* is the number of problems discovered after analyses by n participants

In these five datasets, the number of participants ranged from 15 to 76. Previous studies of these datasets [8, 19, 23, 25] demonstrated that the probability of problem detection was heterogeneous. As suggested by the results of the simulation study, the methods not taking account of heterogeneity considered that the discovery process was complete or very close to being complete for all datasets (except MACERR: see below). Thus, we compared the results of the methods that do account for heterogeneity. It is noteworthy that the estimates of *μ* and *σ*^2^ by both the LNBzt and the matrix-based methods fell within the range observed in our simulation study for all datasets other than MACERR.

All five methods considered that the SAVINGS and MANTEL datasets were complete after 34 and 76 participants had been included, respectively. However, the confidence intervals produced by the matrix-based and the LNBzt methods suggest that few problems had yet to be discovered.

The matrix-based and the LNBzt methods estimated similar number of problems for EDU3D (${\hat{m}}_{\mathrm{matrix}-\mathrm{based}}=152$ and ${\hat{m}}_{LNBzt}=155\Big)$. The 95% confidence interval was broader for the LNBzt method (132 to 195) than for the matrix-based method (135 to 167).

The infusion pumps in the INFPUMP study were in early-stage development, and an additional re-design phase (for fixing the usability problems discovered) was planned; this explains why n =107 unique problems were detected by the 34 participants in the usability testing. The LNBzt and matrix-based methods gave similar estimates and confidence intervals: ${\hat{m}}_{\mathrm{LNBzt}}=122$ (i.e. 15 undiscovered problems), with a 95% confidence interval from 115 to 131, whereas ${\hat{m}}_{\mathrm{matrix}-\mathrm{based}}=120$, with a 95% confidence interval from 112 to 143. The parameters computed by the matrix-based method predicted an average probability of detection ${\hat{\mu}}_{\mathrm{matrix}-\mathrm{based}}= logit(0.136)$ and a dispersion of ${\hat{\sigma}}_{\mathrm{matrix}-\mathrm{based}}=1.52$. For the LNBzt method, the probability ${\hat{\mu}}_{\mathrm{LNBzt}}= logit(0.136)$ was the same, and the dispersion was slightly higher (${\hat{\sigma}}_{\mathrm{LNBzt}}=1.50$). The confidence interval (from 110 to 136) was narrower. The true number of problems with the pump was not known because it was redesigned after 34 participants had tested the device. However, if we accept the parameters $\hat{\mu}$ and *σ* as true and apply the results of our simulation study, the INFPUMP data suggest that the LNBzt and matrix-based methods are both reliable. Nevertheless, the breadth of the respective confidence intervals emphasizes the remaining uncertainty for these two methods.

Using the MACERR data, the LNBzt predicted a very low average probability of detection (${\hat{\mu}}_{\mathrm{LNBzt}}= logit(0.014)$) and a high level of heterogeneity (${\hat{\sigma}}_{\mathrm{LNBzt}}=1.90\Big)$. These values were out of the range of the settings tested in the simulation study, and suggested that the number of “rare” problems was high. This might explain the high number of problems predicted by the LNBzt method (${\hat{m}}_{\mathrm{LNBzt}}=449$), and the very large 95% confidence interval (from 256 to 1301). The matrix-based method’s estimate was lower (${\hat{m}}_{Matrix- based}=382$), and the 95% confidence interval was narrower (346 to 440). However, the number of participants included in MACERR was low (*n* = 15); a larger number of participants would have been necessary to discover new problems and improve the estimates.

On average, computation of the estimate and its confidence interval took less than 10 min for the matrix-based method, less than 1 min for the LNBzt method, and only a few seconds for the three other methods.

## Discussion

We decided to model the full discovery matrix (including unobserved columns) and not just a summary of the data (e.g. the margins). The estimation problem was considered simultaneously in terms of the (heterogeneous) probability of problem detection and the number of problems. Although the experimental conditions in real-life usability testing are unknown, the matrix-based method outperformed the other methods and appeared to be the most reliable in a broad range of settings.

Most of the currently available methods assume that the probability of detection is the same for all problems. This assumption is likely to be wrong, since real data show that the probability of detection varies [19, 23]. Furthermore, ignoring heterogeneity is known to strongly bias the results [24, 39]. We therefore developed a method that accounted for heterogeneity in the probability of problem discovery *p*; we used a logit-normal distribution as a plugin to model this uncertainty. The choice of this distribution was convenient in that it allowed us to compare our method with the only published model that accounts for heterogeneity. However, there are no data for confirming the validity of this choice. Nevertheless, this limitation could be easily overcome by replacing the logit-normal by another distribution (such as beta or gamma) if it proves to be more appropriate. This choice could be made using model choice criteria (e.g. the Akaike information criterion or the Bayesian information criterion). However, it should be borne in mind that for a small sample size, fitting for both incompleteness and heterogeneity is complex and inevitably leads to a high degree of uncertainty.

Here, we sampled *μ* and *σ* for fixed values of *m*. This turned out to be a rather time-consuming strategy because we had to run as many chains as there were values of m. We chose not to sample directly from the joint distribution $P\left(\mu, \sigma, m|\mathbbm{d}\right)$ because the dimension of the latent parameters *p*_1_, *p*_2_, …, *p*_*m*_ varied as a function of *m* - making it impossible to use a standard MCMC algorithm. In this particular situation, use of the reversible jump algorithm [40] might be a solution but would considerably complicate our algorithm.

There are two key moments in medical device development for assessing the best method. Early in the development cycle, the device is not mature; usability testing is referred to as “formative” because many usability problems are being discovered and corrected in an iterative design improvement process. Just before market access, usability testing is referred to as “validation” testing; they are performed on the final version of the device to ensure that no critical usability problems remain [1, 2].

The number of participants in the validation testing is an important parameter for both the regulatory authorities and the device manufacturer. Indeed, a sufficient sample size will (i) guarantee the medical device’s compliance with the safety standards required for market authorization, and (ii) avoid a “black swan” effect that would strongly affect the manufacturer’s credibility and profitability [41]. The validation testing focuses on the detection of infrequent usability problems. The US Food and Drug Administration requires a minimum of 15 participants [1]. This minimum is based on a naïve estimate, which has been proven to dramatically underestimate the true number of usability problems for this number of participants [12]. Indeed, the average coverage probability observed in our simulation study for *n* = 20 was as low as 12% and did not exceed 51%. Furthermore, this threshold does not consider heterogeneity in the probability of problem detection. Our findings suggest that to produce a relevant estimate with the matrix-based method, at least 20 participants are required in the validation step. In fact, the matrix-based method displayed good statistical properties with as few as 20 participants.

Since the validation testing only concerned problems that are probably less frequent, one could question the need to use methods that account for a heterogeneous probability of problem detection. In fact, problems are expected to be “homogeneously rare”. To the best of our knowledge, however, the assumption of homogeneity for rare problems has no theoretical or experimental basis. Furthermore, human factor engineers will define the usability testing’s experimental conditions according to the risk analysis, in order to facilitate the detection of problems previously described in the literature. If an engineer suspects the existence of problem removing the cap from an adrenaline pen, he/she might choose to evaluate the device in a more realistic test environment (e.g. with an actor pretending to go into anaphylactic shock); the problem is more likely to occur there than in a quiet, low-fidelity environment. By making some problems more detectable, the human factor engineer might introduce a degree of heterogeneity into the discovery process.

The choice of method was even more obvious for “formative” testing. In our simulations, the “formative” testing corresponds to a setting in which usability problems are frequent and numerous. Schmettow’s usability testing of a medical infusion pump is also an example of a formative assessment because it was followed by a redesign. Here, we proved that matrix-based methods are more reliable and have low bias and high consistency. As in the case of the infusion pump, a reliable estimate from a small number of participants is an economic advantage for the manufacturer, who can shorten redesign cycles, accelerate device development, and hasten market access. The matrix-based method met this requirement because it required the fewest participants to guarantee good statistical properties. Another strength of the matrix-based method is its ability to embed previous knowledge through the prior parameters. Indeed, we used weakly informative priors for *μ* and *σ* to avoid introducing information that we did not have about the medical device in question. However, one could take advantage of prior knowledge from earlier stages in device development or from a formative usability assessment to increase the accuracy of the estimate, especially when the sample size is small (i.e. an early control strategy). This approach is actually encouraged by regulatory bodies for medical device clinical trials [42] and helps to reduce the overall sample size.

Although we have suggested a threshold of 20 participants as the minimum sample size for obtaining a reliable estimate with the matrix-based method, we do not consider this to be the final threshold or a “magic number”. Indeed, as suggested by various researchers, the estimation models should be run iteratively as the sample size increases [4]. Thus, estimation models constitute a means of controlling and ensuring quality in formative testing and should not solely be considered as a checkpoint for validation testing. Although the matrix-based method was more reliable, the LNBzt method could be used to double check the estimates - especially when high dispersion and/or the presence of very rare problems is suspected. Indeed, the LNBzt method’s coverage probability is high, and the overestimation bias makes it a conservative method that could usefully prevent the usability testing from being stopped too early.

## Conclusions

Estimation models (and particularly matrix-based models) are of value in estimating and monitoring the detection process during usability testing. Matrix-based models have a solid mathematical grounding and, with a view to facilitating the decision-making process for both regulators and device manufacturers, should be incorporated into current standards. To this end, the step-by-step tutorial provided here should facilitate the practical use of the matrix-based method in the evaluation of medical devices.

## Supplementary information

**Additional file 1. **Step by step instructions for the matrix-based method presented in this manuscript. Open the file *“tutorial.pdf*” and follow the instructions.

**Additional file 2. **Tabulated version of the results of the simulation study presented in the main manuscript. S-Table 5: Bias in the prediction of m: the mean error (as a percentage of the true m) as a function of the sample size (n). S-Table 6: Consistency in the prediction of m: the RMSE for the prediction of m as a function of the sample size (n). S-Table 7: Coverage probability (in % of the 95% confidence interval) of $\hat{m}$ with each combination (*m*, *μ*, *σ*, *n*).

## Data Availability

The data and code supporting the conclusions of this article are available in the GitHub repository, https://zenodo.org/badge/latestdoi/279117812.

## Abbreviations

LNBzt: Logit normal binomial zero truncated
GT: Good-Turing

## References

[1] US-FDA (2016). Applying human factors and usability engineering to medical devices: Guidance for industry and Food and Drug Administration staff.

[2] UK-MHRA (2017). Human Factors and Usability Engineering – Guidance for Medical Devices Including Drug-Device Combination Products. In*.* Edited by Agency MHpR.

[3] US-FDA (2012). Medical device recall report FY2003 to FY2012. Center for Devices and Radiological Health.

[4] Borsci S, Macredie RD, Barnett J, Martin J, Kuljis J, Young T (2013). Reviewing and extending the five-user assumption: a grounded procedure for interaction evaluation. ACM Transactions on Computer-Human Interaction (TOCHI).

[5] Borsci S, Macredie RD, Martin JL, Young T (2014). How many testers are needed to assure the usability of medical devices?. Expert Rev Med Devices.

[6] Lewis JR (1994). Sample sizes for usability studies: additional considerations. Hum Factors.

[7] Kanis H (2011). Estimating the number of usability problems. Appl Ergon.

[8] Lewis JR (2001). Evaluation of procedures for adjusting problem-discovery rates estimated from small samples. Int J Human-Computer Interaction.

[9] Hertzum M, Jacobsen NE (2003). The evaluator effect: a chilling fact about usability evaluation methods. Int J Human-Computer Interaction.

[10] Schmettow M (2012). Sample size in usability studies. Commun ACM.

[11] Borsci S, Londei A, Federici S (2011). The bootstrap discovery behaviour (BDB): a new outlook on usability evaluation. Cogn Process.

[12] Faulkner L (2003). Beyond the five-user assumption: benefits of increased sample sizes in usability testing. Behav Res Methods Instrum Comput.

[13] Lewis JR (2000). Using discounting methods to reduce overestimation of p in problem discovery usability studies. In: Citeseer.

[14] Sauro J, Lewis JR (2016). Quantifying the user experience: practical statistics for user research: Morgan Kaufmann.

[15] Thomas DG, Gart JJ (1971). Small sample performance of some estimators of the truncated binomial distribution. J Am Stat Assoc.

[16] Virzi RA (1992). Refining the test phase of usability evaluation: how many subjects is enough?. Hum Factors.

[17] Nielsen J, Landauer TK (1993). A mathematical model of the finding of usability problems. Proceedings of the INTERACT'93 and CHI'93 conference on Human factors in computing systems: 1993.

[18] Good IJ (1953). The population frequencies of species and the estimation of population parameters. Biometrika.

[19] Schmettow M. Controlling the usability evaluation process under varying defect visibility. In: Proceedings of the 23rd British HCI Group Annual Conference on People and Computers: Celebrating People and Technology: 2009: British Computer Society; 2009. p. 188–97.

[20] Finney D (1947). The truncated binomial distribution. Ann Eugenics.

[21] Rider PR (1955). Truncated binomial and negative binomial distributions. J Am Stat Assoc.

[22] Shah S (1961). The asymptotic variances of method of moments estimates of the parameters of the truncated binomial and negative binomial distributions. J Am Stat Assoc.

[23] Schmettow M: Heterogeneity in the usability evaluation process. *People and Computers XXII Culture, Creativity,* Interaction 22 2008:89–98.

[24] Caulton DA (2001). Relaxing the homogeneity assumption in usability testing. Behav Inform Technol.

[25] Schmettow M, Vos W, Schraagen JM (2013). With how many users should you test a medical infusion pump? Sampling strategies for usability tests on high-risk systems. J Biomed Inform.

[26] DasGupta A, Rubin H (2005). Estimation of binomial parameters when both n, p are unknown. J Stat Planning Inference.

[27] Fisher RA (1941). The negative binomial distribution. Ann Eugenics.

[28] Haldane JB (1941). The fitting of binomial distributions. Ann Eugenics.

[29] Carroll RJ, Lombard F (1985). A note on N estimators for the binomial distribution. J Am Stat Assoc.

[30] Olkin I, Petkau AJ, Zidek JV (1981). A comparison of n estimators for the binomial distribution. J Am Stat Assoc.

[31] Hall P (1994). On the erratic behavior of estimators of N in the binomial N, p distribution. J Am Stat Assoc.

[32] Robert C (2007). The Bayesian choice: from decision-theoretic foundations to computational implementation: Springer Science & Business Media.

[33] Meng X-L, Wong WH. Simulating ratios of normalizing constants via a simple identity: a theoretical exploration. Stat Sin. 1996:831–60.

[34] Bach C, Scapin DL (2010). Comparing inspections and user testing for the evaluation of virtual environments. Intl J Human–Computer Interaction.

[35] Lewis JR, Henry SC, Mack RL (1990). Integrated office software benchmarks: a case study. In: Interact.

[36] Nielsen J, Molich R (1990). Heuristic evaluation of user interfaces. Proceedings of the SIGCHI conference on Human factors in computing systems.

[37] Team SD (2018). RStan: the R Interface to Stan. R package version 2.17. 3. In.

[38] Gronau QF, Singmann H, Wagenmakers E-J (2017). Bridgesampling: An R package for estimating normalizing constants. arXiv preprint arXiv:171008162.

[39] Woolrych A, Cockton G (2001). Why and when five test users aren’t enough. Proceedings of IHM-HCI 2001 conference: 2001: Eds.

[40] Green PJ (1995). Reversible jump Markov chain Monte Carlo computation and Bayesian model determination. Biometrika.

[41] Bias RG, Mayhew DJ (2005). Cost-justifying usability: an update for the internet age: Elsevier.

[42] US-FDA (2010). Guidance for the use of Bayesian statistics in medical device clinical trials.

