# Supplementary material for: Estimating the number of usability problems affecting medical devices: modelling the discovery matrix
Source: BMC Med Res Methodol. 2020 Sep 18;20:234. doi: 10.1186/s12874-020-01091-y (PMC7653970; doi:10.1186/s12874-020-01091-y)
Supplement: Supplementary file 1 — Additional file 1. Step by step instructions for the matrix-based method presented in this manuscript. Open the file “tutorial.pdf” and follow the instructions. [file 12874_2020_1091_MOESM1_ESM.pdf]

Step-by-step instructions for applying the matrix-based method

A. Install R

R is a programming language and free software environment for statistical computing supported by the R Foundation for Statistical Computing. It can be installed via the CRAN http://cran.R-project.org.

B. Install the rstan and bridgesampling packages

Run the following code:

```
> install.packages("rstan")
> install.packages("bridgesampling")
```

C. Load the discovery matrix

Consider the d.csv file, a discovery matrix of 0 and 1 (saved in .csv format). It can be imported into R via the following command line instruction:

```
> d <- read.csv("d.csv")
```

D. Load the stan model

Load the rstan library and compile the stan model (the file *draw_mu_s2.stan* is provided in Additional file 3):

```
> library(rstan)
> model <- stan_model("draw_mu_s2.stan")
```

E. Run the matrix-based method

The matrix-based approach is implemented in the file *functions.R* (provided in Additional file 3). This file can be sourced using the following command line:

```
> source("functions.R")
```

The *heterogenous_bayes()* function runs the matrix-based method on the discovery matrix and returns the estimated m (M is the maximum number of problems on the m grid):

```
> res <- heterogeneous_bayes(d, M = 50, full_output = FALSE)
> print(res)
```

In order to obtain more details (e.g. the posterior distribution of m and sampled values of μ and σ^2 given m), the full_output argument must be set to TRUE:

```
> res_full <- heterogeneous_bayes(d, M = 50, full_output = TRUE)
> plot(res_full$posterior_m,type = "h", main = "Posterior distribution p(m|d)")
> head(res_full$simu_mu_s2)
```

Data

Copy the following text in a *d.csv* file or directly download it from GitHub:

https://github.com/alexandre-caron/matrix_based-usability/blob/master/tutorial/d.csv

```
"V1","V2","V3","V4","V5","V6","V7","V8","V9","V10","V11","V12","V13","V14","V15","V16",
"V17","V18","V19","V20","V21","V22","V23","V24","V25","V26","V27"
0,0,1,0,0,0,1,0,0,0,1,1,1,0,0,0,0,0,0,0,0,1,0,0,0,0
0,1,0,0,0,0,0,1,0,0,1,1,0,0,0,0,0,1,0,0,0,0,0,0,0,0,0
1,0,0,0,0,0,0,0,0,0,0,1,0,0,1,0,0,1,1,0,1,0,1,0,0,0,0
0,0,0,0,0,0,0,0,0,0,0,0,0,1,0,0,0,0,0,0,0,0,0,0,1,0,0
1,0,0,0,0,0,0,0,1,1,1,0,0,0,0,0,0,0,0,0,0,0,0,0,0,0,0
0,1,0,0,0,0,0,1,0,0,0,0,1,0,0,0,0,0,0,0,1,0,1,0,0,0,0
1,0,0,0,1,0,0,0,0,0,1,1,1,0,1,0,0,1,0,1,1,1,1,1,0,0,0
0,0,0,0,0,0,0,0,1,0,0,0,0,0,0,0,0,1,1,0,0,0,0,0,0,1,0
0,0,0,0,0,0,1,0,0,0,1,0,1,0,0,0,0,0,0,1,1,0,1,0,0,0,1
1,0,0,0,0,0,0,0,0,0,1,0,0,0,1,0,0,1,0,0,1,0,0,0,0,1,0
0,0,0,0,0,0,0,0,0,0,0,0,0,0,0,0,0,1,0,0,0,1,0,1,0,0,0,0
0,1,0,1,0,1,0,0,0,0,1,1,0,0,1,0,0,0,0,1,0,0,0,1,0,1,0
0,0,1,0,1,0,1,1,0,0,1,0,0,0,0,1,0,1,0,1,0,0,0,0,0,1,0
0,1,0,0,0,0,1,0,0,0,0,0,1,0,0,0,0,1,0,0,1,0,1,0,0,0,0
0,0,0,0,1,0,0,0,1,1,0,1,0,0,0,0,0,0,0,0,0,0,1,0,0,0,0
```

Stan Model

Copy the following code in a *draw_mu_s2.stan* file or directly download it from GitHub:

https://github.com/alexandre-caron/matrix_based-usability/blob/master/functions/draw_mu_s2.stan

```
// Stan code for sampling mu and s2 given m and the data x[m]
// Sampling of m is still a problem ...
data {
  int<lower=0> n;        // number of subjects
  int<lower=0> m;        // number of problems
  int<lower=0> x[m];     // number of dectection of each problem

  // hyperparameters
  real mu_prior_mean;
  real<lower=0> mu_prior_sd;
  real<lower=0> s2_prior_a;
  real<lower=0> s2_prior_b;
}
parameters {
  real mu;               // mean of the logit_p
  real<lower=0> s2;      // variance of logit_p
  real logit_p[m];       // vector of logit_p
}
model {
  target += normal_lpdf(mu | mu_prior_mean, mu_prior_sd);    // prior for mu
  target += inv_gamma_lpdf(s2 | s2_prior_a, s2_prior_b);  // prior for s2
  for (j in 1:m) {
    target += normal_lpdf(logit_p[j] | mu, sqrt(s2)); // logit_p given mu and s
    target += binomial_lpmf(x[j] | n, inv_logit(logit_p[j]));   // log-likelihood of the data
  }
```

Functions

Copy the following code in a *functions.R* file or directly download it from GitHub:

https://github.com/alexandre-caron/matrix_based-usability/blob/master/functions/functions.R

```
# Required packages
library(rstan)
library(bridgesampling)

model <- stan_model('draw_mu_s2.stan') # compile the Stan program

# Input :
# - d : a discovery matrix
# - M : the maximal number of problem
# - h : the number of iterations to break after if no improvement of the
# likelihood has been produced (default = 5)
# - ... : some optional inputs as the hyperparameters
# Ouput :
# - return the maximum a posteriori estimator of m (default), or posterior
# distribution of m and resulting posterior sample of p(mu, s2|d, m) if
# full_output = TRUE
heterogeneous_bayes <- function(d, M, h = 5, mu_prior_mean = 0, mu_prior_sd = 10000,
                                s2_prior_a = 0.5, s2_prior_b = 0.5, full_output = FALSE) {
  j = ncol(d)
  n = nrow(d)
  nl = colSums(d)
  if (full_output){
    simu = NULL
  }
  lp = rep(-Inf, M)
  for (k in j:M){
    # print(paste("k = ",k))
    x <- c(nl, rep(0, k - j)) # pad the vector with 0 for undiscovered problems
    usability_dat <- list(n = n, m = k, x = x, mu_prior_mean = mu_prior_mean,
                          mu_prior_sd = mu_prior_sd, s2_prior_a = s2_prior_a,
                          s2_prior_b = s2_prior_b)
    fit <- sampling(object = model,  data = usability_dat, refresh = 0)
    # fit <- stan(file = 'draw_mu_s2.stan', data = usability_dat,
    #         verbose = FALSE, open_progress = 0)
    lp[k] <- bridge_sampler(fit,silent = TRUE)$logml
    lp[k] <- lp[k] + lchoose(k,j)
    # print(paste0(k, "-",lp[k]))
    # It is eventually possible to get values of mu and s2 given m
    # by removing the 1000 first warmup iterations
    # But the resulting object can eventually be very big
```

```
    if (full_output){
      mu = do.call("c", lapply(fit@sim$samples, function(x) x$mu[1001:2000]))
      s2 = do.call("c", lapply(fit@sim$samples, function(x) x$s2[1001:2000]))
      simu = rbind(simu, cbind(mu = mu, s2 = s2, m = k))
    } else {
      # If no amelioration during the h last iteration => stop
      if ((k >= (h+j)) & (lp[k - h] > max(lp[(k - h + 1):k]))){
        k = k - h
        break
      }
    }
  }
  if (full_output){
    lp <- lp - max(lp)
    posterior_m <- prop.table(exp(lp))
    posterior_m <- cbind(m = 1:M, posterior = posterior_m)
    return(list(posterior_m = posterior_m, simu_mu_s2 = simu))
  } else {
    return(k)
  }
}
```
